# Study of Cu Electrochemical Polishing Mechanism With Observation of Water Acceptor Diffusion

**DOI:** 10.3389/fchem.2021.763508

**Published:** 2021-10-21

**Authors:** Kimoon Park, Jinhyun Lee, Youjung Kim, Sangwha Yoon, Bongyoung Yoo

**Affiliations:** ^1^ Department of Advanced Material Science & Engineering, Hanyang University, Ansan-si, South Korea; ^2^ Department of Materials Engineering, Hanyang University, Ansan-si, South Korea; ^3^ Department of Material Science and Chemical Engineering, Hanyang University, Ansan-si, South Korea

**Keywords:** electropolishing, copper, water acceptor, scanning electrochemical microscopy, diffusion

## Abstract

The salt-film and water acceptor mechanisms were generally accepted mechanisms for Cu electrochemical polishing (ECP) theory. These mechanisms of Cu ECP are still controversial for a long time. Conventional and new electrochemical analysis methods were used to investigate the mechanisms and behaviors of Cu electrochemical polishing. Two cases of Cu dissolution, with and without polishing, were classified by results of linear scan voltammetry (LSV) and scanning electron microscopy (SEM). The electrochemical impedance spectroscopy (EIS) results showed the main difference in these two cases was in the low-frequency region. However, it was hard to distinguish between the salt-film and water acceptor mechanisms by conventional electrochemical analysis. A scanning electrochemical microscopy (SECM) system, a new electrochemical analysis method that measures the electrolysis currents of the water acceptors along with a set distance from the substrate, was used to investigate the Cu ECP mechanism. Accordingly, the diffusion of the water acceptors was successfully confirmed for the first time. Finally, the mechanisms of the Cu ECP are definitively described by using all analysis results.

## Introduction

Cu electrochemical polishing (ECP) is an electrochemical process that makes a Cu surface smooth when Cu electrodes are anodically polarized in the ECP electrolytes. This Cu ECP method has several advantages, such as a solution-based process that uses simple equipment, selectivity for the conductive substrate, and causing no mechanical damages. Due to these unique properties, the Cu ECP process can be used in various applications that treat surfaces for cosmetic purposes (Du and Suni, 2004), for substrates for graphene growth (Zhang et al., 2012), for TEM and EBSD samples (Sun et al., 2005; Lapeire et al., 2013), and for Cu planarization in semiconductor interconnections (Chang et al., 2002; Chang et al., 2003; Padhi et al., 2003; Liu et al., 2005; Suni and Du, 2005; West et al., 2005; Liu et al., 2006; Liu et al., 2006). Despite the many potentials of Cu ECP, its actual mechanisms are still controversial.

The simple mechanism of Cu ECP in concentrated phosphoric acid solutions was first suggested by Jacquet’s viscous film theory (Jacquet, 1936). A bluish viscous layer forms on the Cu substrate and the thickness of this layer is different between its protrusions and valleys. This difference means that the resistances at the protrusions are different from those in the valleys, and therefore ECP occurs. This theory was challenged by Elmore’s diffusion theory (Elmore, 1939; Elmore, 1940), wherein the thicknesses of the diffusion layer of Cu ions from the Cu surface to the bulk electrolyte are different at the protrusions and in the valleys. These different diffusion layer thicknesses affect the limiting current of Cu dissolution reaction. The limiting current is higher at the protrusions, while that is lower at the valleys. Therefore, these phenomena cause the ECP. Then, in contrast to this theory, Edwards (1953) proposed acceptor diffusion theory wherein the ECP is governed by the diffusion of anionic acceptors such as (PO_4_)^3−^, (HPO_4_)^2−^, and (H_2_PO_4_)^−^ into the Cu surface from the bulk electrolyte. The ECP rates are dependent on the diffusion of the acceptors into the Cu surface; the diffusion rate is high at the protrusions and low in the valleys.

After these mechanisms were thoroughly evaluated, the salt-film mechanism and the water acceptor mechanism came to be considered the main processes that explained the behavior of Cu ECP. Landolt. (1987) first described the salt-film mechanism, whereby salts are precipitated when the concentration of metal ions on the Cu surface produced by the dissolution reaction exceeds the solubility limit; the precipitated salt film forms on the Cu surface. Due to this salt film, metal dissolution is limited by the diffusion of metal ions from the film to the bulk electrolyte. In contrast, the water acceptor mechanism is similar to Edward’s acceptor diffusion theory except that the acceptor is water instead of anionic acceptors. And unlike in the salt-film mechanism, the most important factor for the polishing effect is not the diffusion of Cu ions into the bulk electrolyte, but, rather, the diffusion of water acceptors into the Cu surface.

Between these two mechanisms, the water acceptor mechanism has been more accepted by electrochemical impedance spectroscopy (EIS) studies (Glarum and Marshall, 1985a, Glarum and Marshall, 1985b; Vidal and West, 1995a, Vidal and West, 1995b), which indicated that the water depletion layer acts as a viscous film, so the mass transfer control reaction of the water acceptors becomes a critical factor. Wagner. (1954) mathematically analyzed an ideal Cu ECP process based on the water acceptor mechanism, and various other studies proposed mechanisms to explain Cu ECP behavior (Hoar and Rothwell, 1964; Kojima and Tobias, 1973; Pointu et al., 1981; Pointu et al., 1983a, Pointu et al., 1983b). The water acceptor mechanism has remained the most accepted based on previous studies. However, Cu ECP mechanisms are still controversial because the ECP process is complicated, which is many factors such as metal ion, anions, and acceptors affect the process. Therefore, new analyses are required for the direct explanation and confirmation of Cu ECP behavior.

Accordingly, in this paper we report on the mechanisms and behaviors of Cu ECP using the conventional electrochemical analysis methods and a new electrochemical analysis method. So, the electrochemical behaviors of Cu ECP at various ECP potentials were compared and studied. In addition to these conventional analyses, quantities of water at various distances from the Cu substrate were estimated by measuring the water electrolysis reaction currents using a scanning electrochemical microscopy (SECM) system for the direct observation and verification of Cu ECP behavior based on the water acceptor mechanism. Based on the data, we here discuss the mechanisms, behaviors, and critical factors of Cu ECP.

## Materials and Methods

We first conducted typical analysis and experiments in a 100 ml cell with a conventional three-electrode system. The three-electrode system consisted of an electrodeposited Cu substrate as the working electrode, a Pt-coated Ti plate as the counter electrode, and a saturated Ag/AgCl electrode (3M KCl saturated) as the reference electrode. For preparing the working electrodes, Ti (20 nm)/Cu (200 nm) seed layers were deposited on an Si wafer by the evaporation method, and Cu film was galvanostatically electrodeposited at −50 mA/cm^2^ for 300 s on Si/Ti (20 nm)/Cu (200 nm) substrates with an area of 1 × 1 cm^2^.

Reagent-grade chemicals were utilized for the all of the Cu electrodepositions and Cu ECP experiments. The electrolytes for Cu electrodeposition consisted of 1.0 M copper sulfate (CuSO_4_, 99.5%, YAKURI, Japan), 0.58 M sulfuric acid (H_2_SO_4_, 95%, Daejung Chemicals & Metals, Korea), and 1.9 mM hydrochloric acid (HCl, 35%, Daejung Chemicals & Metals, Korea) in 100 ml of 18.6 MOhm deionized water. 85% phosphoric acids (H_3_PO_4_) were utilized as electrolytes for the Cu ECP.

Conventional electrochemical analysis, deposition, and polishing were carried out using a potentiostat/galvanostat (VersaSTAT 4, AMETEK Inc., United States). For analysis of electrochemical behavior and appropriate ECP potential selection, LSV analysis was conducted at the potential range between the open circuit potential (OCP) to 2.5 V vs Ag/AgCl with a scan rate of 10 mV/s without agitation. After selection of the ECP potentials at 0.25, 0.375, 0.50, 0.90, and 1.30 V, Cu substrates were electrochemically polished at the potential of 1.3 V in 85% phosphoric acid without agitation. The surfaces of the electrochemically polished Cu substrates were observed using field emission scanning electron microscopy (FESEM; MIRA3, TESCAN Orsay Holding, a.s., Czech Republic). EIS analysis was also conducted at the selected ECP potentials with an amplitude of 10 mV in the frequency range of 50 kHz to 10 Hz so as to understand the Cu ECP behavior. EIS and SECM were performed after “preconditioning” that applying the selected potential for 60 s to the Cu substrates in phosphoric acid to establish a steady-state interface condition on the Cu substrates (Grimm et al., 1992).

**FIGURE 1:**
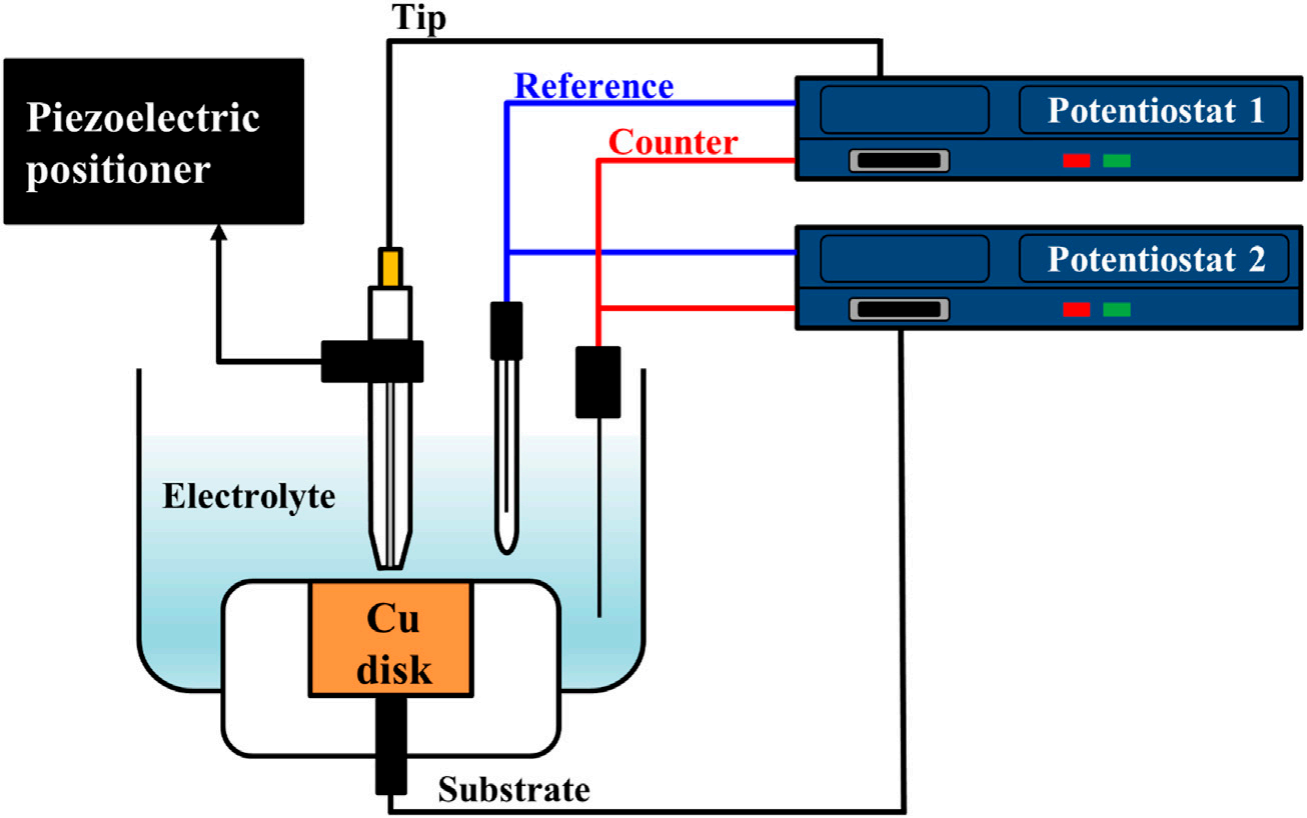
Scheme of cell configuration for water electrolysis current measurement using SECM system.

**FIGURE 2:**
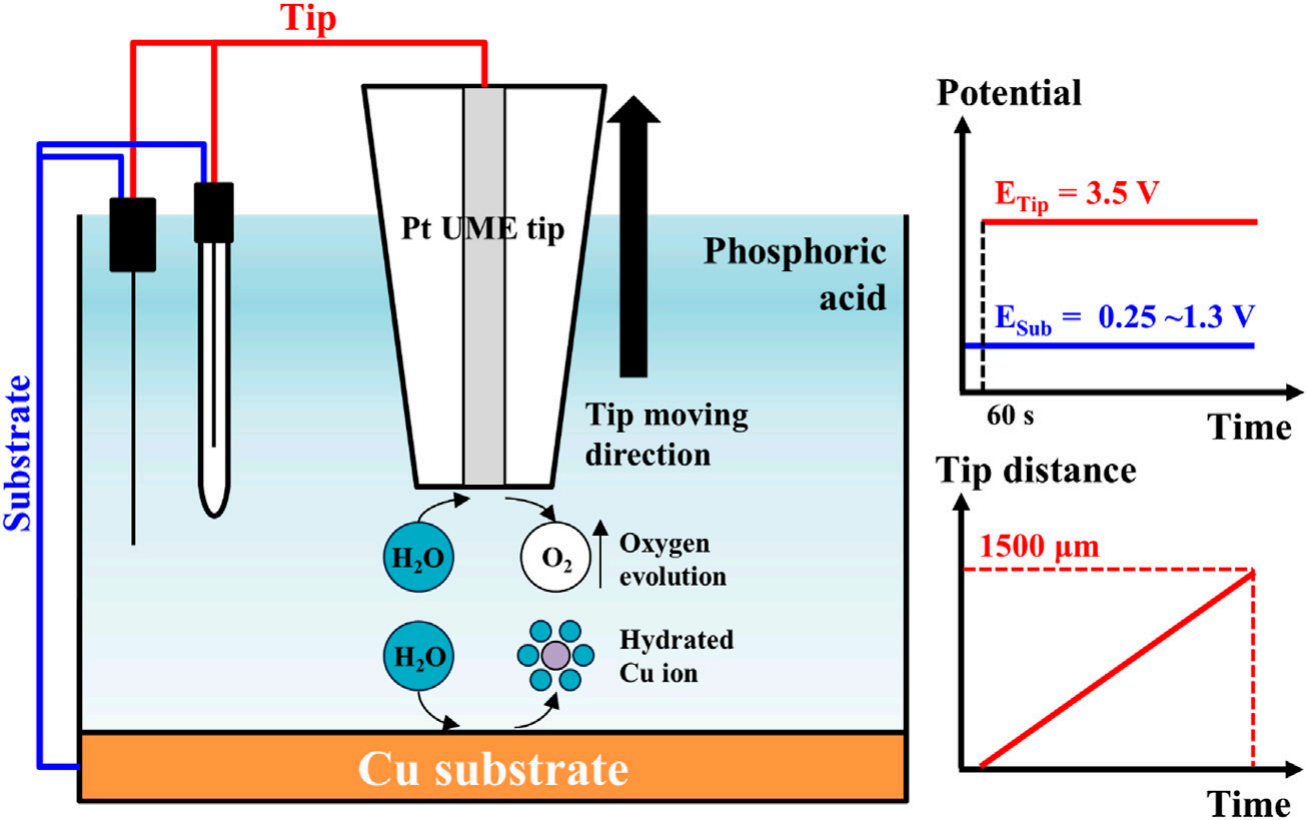
Measurement scheme of electrode reactions for water electrolysis current measurement in SECM system. The Cu ions were dissolved from Cu substrate by applied potential from 0.25 to 1.3 V. The water molecules hydrated the dissolved Cu ions. Oxygen evolution occurs on Pt tip by applied potential 3.5 V after preconditioning for 60 s. A Pt tip moved with 5 μm/s of scan speed.

To estimate the water acceptor quantities along various distances from the Cu substrate, electrolysis currents were measured with the SECM system’s electrochemical scanning probe tool. The cell configuration for this analysis is illustrated in Figure 1. The electrodes consisted of the Pt tip probe (10 μm diameter) and the Cu substrate for the working electrode, a Pt wire for the counter-electrode, and Ag/AgCl electrode (3 M KCl saturated) for the reference electrode. The Pt tip probe and the Cu substrate were arranged with each potentiostat as working electrodes. These configurations were identical with typical configuration of SECM analysis. Based on the configuration of the electrodes, the solutions and the reactions for the analysis are illustrated in Figure 2. The 85% phosphoric acid was utilized to analyze the solutions, and the process details are as follows. First, when the potential of the Cu substrate was applied to 0.25∼1.3 V, the Cu dissolution reaction occurred at the surface of the Cu substrate with these two-step reactions (Matlosz et al., 1994; Han and Fang, 2019):

$$\mathrm{Cu}\to Cu_{\mathrm{ad}}^{2+}\mathrm{+2}e^{-}$$ (1)

$$Cu_{\mathrm{ad}}^{2+}\mathrm{+6}{Η}_{2}O\to {\left[\mathrm{Cu}{\left(H_{2}O\right)}_{6}\right]}^{2+}$$ (2)

After 60 s of preconditioning time, the potential of the Pt tip probe was applied to 3.5 V at the Cu substrate to achieve steady-state for the water acceptor diffusion. The oxygen evolution reaction from the decomposition of the water occurred at the surface of the Pt tip probe with following reaction:

$$2H_{2}O\to O_{2}↑\mathrm{+4}{Η}^{+}\mathrm{+4}e^{-}$$ (3)

These water electrolysis currents were dependent on the water acceptor quantities, and these currents were measured at various Pt tip distances from the Cu substrate. The initial tip distance from the Cu substrate was set to 1 μm to prevent direct current from passing current between the Pt tip and the Cu substrate; then the tip distance was varied from 1 μm to 1,500 μm with 5 μm/s of scan speed. All electrochemical analyses were performed at 20°C.

## Results and Discussion

**FIGURE 3:**
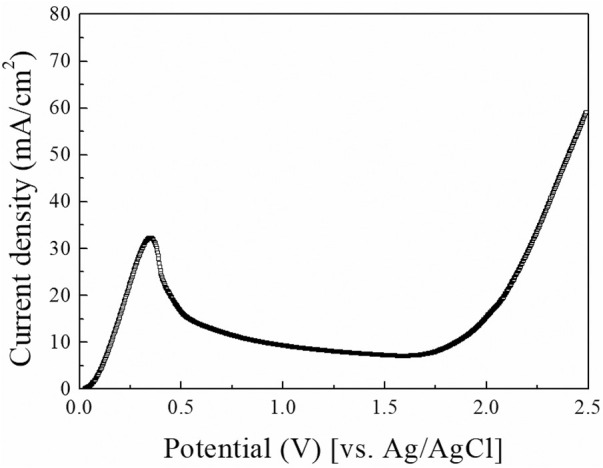
Linear scan voltammogram of Cu substrate in 85% phosphoric acid. A sweep was done with a scan rate of 10 mV/s.

LSV is a fundamental and powerful analysis method to study the electrochemical behavior of Cu ECP. Therefore, we first conducted LSV analysis in 85% phosphoric acid, and the resulting the voltammogram is shown in Figure 3. At the initial potential range, anodic current began to increase until 0.375 V. According to Shieh et al. (2004), these anodic current in this potential range are related to the direct dissolution of Cu, resulting in dully etched surfaces. Over 0.375 V, the anodic current was decreased to 0.50 V. This phenomenon indicates generating a passivation layer on the anodic surface. When the ECP potential was applied over 0.50 V, the current remained nearly constant up to 1.7 V. This current plateau region is generally considered the Cu ECP reaction (Jacquet, 1936; Elmore, 1939; Glarum and Marshall, 1985a; Landolt, 1987; Han and Fang, 2019). After that current plateau, the current was increased again from the potential above 1.7 V by the oxygen evolution reaction (Shieh et al., 2004), which caused severe etch pits.

**FIGURE 4:**
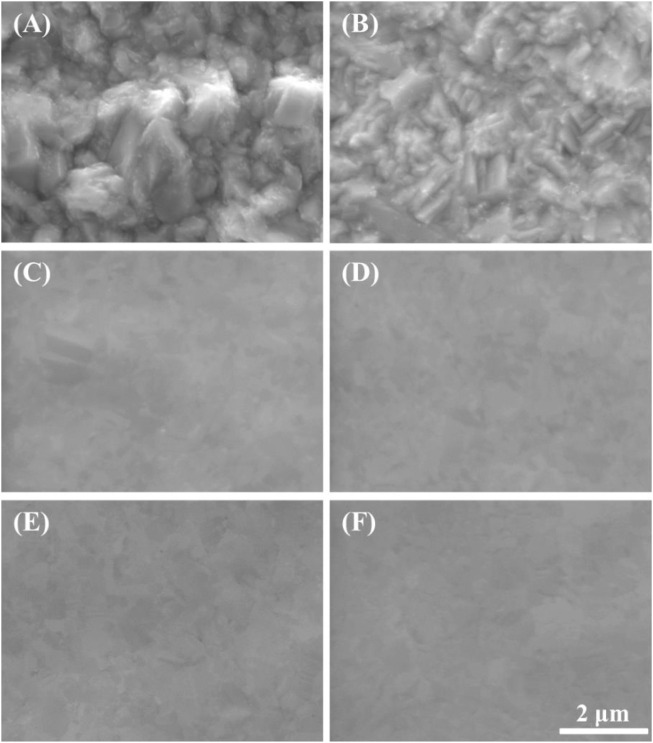
SEM images of anodically polarized Cu substrates in 85% phosphoric acid at various potentials with a charge density of 2.5 C/cm^2^: **(A)** before polarized, **(B)** 0.25 V, **(C)** 0.375 V, **(D)** 0.50 V, **(E)** 0.90 V, and **(F)** 1.30 V.

In addition to interpreting previous papers, we selected five potential points for the exact interpretation of the LSV results: 1) 0.25 V as the potential before the peak point, 2) 0.375 V as the potential at the peak point, 3) 0.50 V as the potential after the peak point, 4) 0.90 V and 5) 1.30 V as the potentials in the plateau region. Then the Cu substrates were anodically polarized at the five potential points with a charge density of 2.5 C/cm^2^; their top SEM images are presented in Figure 4. Compared with the SEM images of Cu substrate before polarized (Figure 4A), the Cu ECP effect was observed at all of the ECP potentials (Figures 4C–F) except at 0.25 V, where dully etched Cu was observed (Figure 4B). These results indicate that the Cu dissolution reaction on Cu substrate or interphase between the Cu substrate and the phosphoric acid began to change as the peak potential. Accordingly, we analyzed the difference in dissolution reactions or interphase conditions between the potential before the peak point and the potentials after the peak point.

Previous researchers reported the two models describing the interface condition at the Cu surface during ECP (Glarum and Marshall, 1985a, Glarum and Marshall, 1985b; Landolt, 1987; Vidal and West, 1995a, Vidal and West, 1995b). One is the salt-film model in which Cu salts are precipitated on the Cu substrate when the Cu dissolution exceeds the dissolution limit of the Cu salts. The other is the water-acceptor diffusion model in which water acceptors are diffused toward the Cu substrate. However, in both models, the ECP mechanism is still controversial. Therefore, to precisely determine the interface condition of the Cu surface during ECP, we conducted EIS analysis at the five potential points described earlier.

**FIGURE 5:**
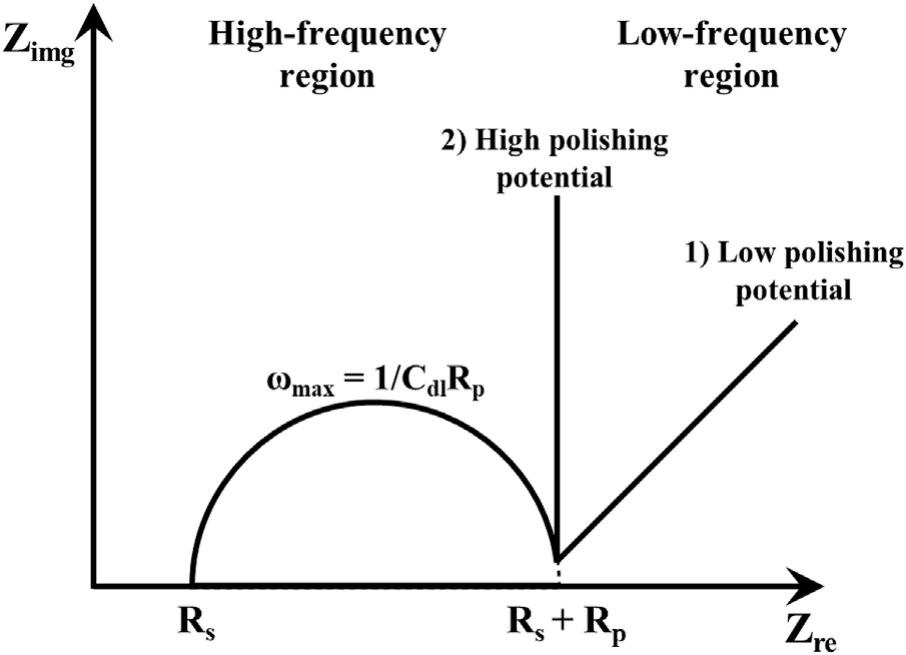
Schematic Nyquist plot according to frequency region.

**FIGURE 6:**
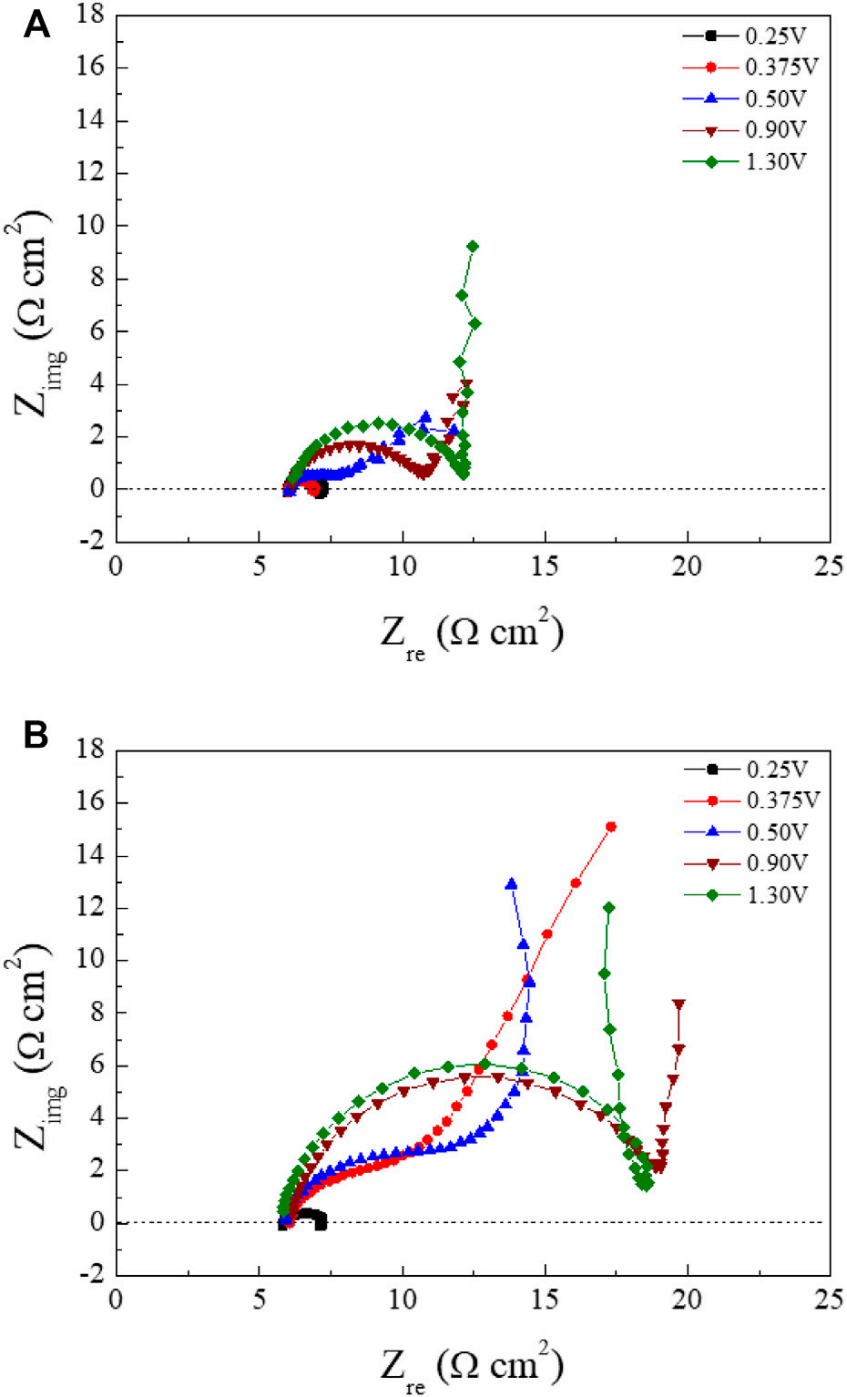
Nyquist plots of the Cu substrates at various polishing potentials in 85% phosphoric acid measured according to preconditioning time **(A)** 0 s and **(B)** 60 s. Frequencies were set on between 50 kHz and 10 Hz.

Figure 5 shows schematic Nyquist plots in the whole frequency range, where R_s_ is the solution resistance from the left intercept of the semicircle, R_p_ is the polarization resistance from the diameter of the semicircle, and C_dl_ is the double layer capacitance calculated from the relation ω_max_ = 1/R_p_C_dl_. ω_max_ represents the frequency when Z_img_ is at maximum value in the semicircle. In the low-frequency region, the angle of the line increased from about 45^o^ to 90^o^ (vertical) as the polishing potentials were increased. This result may be related to the diffusion of the water acceptor and will be discussed later. The Nyquist plots of Cu ECP in the 85% phosphoric acid according to ECP potential were obtained at two preconditioning times, 0 s and 60 s, corresponding with Figure 6A and Figure 6B, respectively.

The fitting results of the Nyquist plots are summarized in Table 1. In the high-frequency region, the R_s_ values were almost consistent with 6.0 Ω cm^2^ regardless of the polishing potential and preconditioning time. These results, which is R_s_ not changed, were evidence of water acceptor diffusion because in the salt-film model the R_s_ changed with the polishing potential (Vidal and West, 1995a). However, the R_p_ was increased, and the C_dl_ was decreased as the anodic potential was increased. These changes of the R_p_ and C_dl_ with the various polishing potentials were different from the EIS results of the water-acceptor mechanism measured by Vidal and West (1995a). Our R_p_ and C_dl_ values changed when the preconditioning times were varied. These changes associated with potentials and preconditioning times can be explained by changes in both the salt-film thickness and the depletion layer thickness in the salt film model and the water acceptor model, respectively (Grimm et al., 1992).

**TABLE 1:** Fitting results of the Nyquist plots according to preconditioning time and applied potentials.

(V)	R_s_ (Ω cm^2^)		R_p_ (Ω cm^2^)		C_dl_ (F/cm^2^)	
	**0 s**	**60 s**	**0 s**	**60 s**	**0 s**	**60 s**
0.25	6.155	6.003	1.110	1.058	7.66 × 10^−5^	1.33 × 10^−4^
0.375	5.947	6.031	0.536	1.903	9.58 × 10^−5^	1.59 × 10^−5^
0.50	6.017	5.964	1.139	3.617	5.76 × 10^−5^	1.42 × 10^−5^
0.90	6.200	6.267	2.36	10.62	1.09 × 10^−5^	7.65 × 10^−6^
1.30	6.139	5.869	2.73	10.69	6.30 × 10^−6^	6.33 × 10^−6^

The low-frequency regions of our Nyquist plots were significantly affected by the potentials and preconditioning times. When the Nyquist plots were obtained as the polishing potentials were applied without preconditioning (Figure 6A), the line in the low-frequency region started to exhibit Warburg impedance from 0.50 to 1.30 V. The angle changed to 90° in low-frequency regions when the polishing potentials exceeded 0.90 V. In the Nyquist plots obtained with the preconditioning time of 60 s (Figure 6B), Warburg impedance was observed from the peak potential, 0.375 V, as seen in Figure 3. When we compared Nyquist plots in the condition of 0.375 V between the preconditioning times of 0 s (Figure 6A) and 60 s (Figure 6B), the Cu ECP occurred when the Cu dissolution reaction was governed by a mass transfer control reaction of the water acceptors. The vertical lines were observed as the anodic potentials exceeded 0.50 V.

These results confirmed that all lines of the low-frequency region were related to the diffusion process due to Warburg impedance. Especially, the vertical lines in the low-frequency region at the high ECP potentials were related to the finite space Warburg (FSW) elements, in which the diffusion condition is a limited diffusion layer and a limited electroactive substance (Oldenburger et al., 2019). This indicated two possible diffusion conditions, one being a porous salt film and the other being a depleted water acceptor layer (Grimm et al., 1992; Matlosz et al., 1994). However, there was no explanation about the low-frequency region in previous reports by Vidal and West. (1995a), Vidal and West. (1995b), leaving it still unclear which mechanism was correct. More analysis was required to interpret these results, so we designed a new analysis method that can directly observe water acceptor diffusion.

To observe the water acceptor diffusion at various distances from the Cu substrate, we measured the water electrolysis current resulting from the oxygen evolution reaction by using the SECM system. The cell configuration of the measurement system and the scheme of the measurement method are presented in Figures 1, 2, respectively. In the three-electrode system, two working electrodes (the Pt tip and the Cu substrate) were installed in the measurement cell. At the Cu substrate, the Cu dissolution reaction occurred when the potentials were anodically applied. At the Pt tip electrode, the water acceptor quantities were estimated by using the oxygen evolution reaction, which was observed in the LSV in Figure 3 when the potentials exceeded 1.7 V. Therefore, the potential at the Pt tip electrode was set to 3.5 V for sufficient electrolysis of the rest of the water acceptors, and the oxygen evolution reaction could occur without dissolution of the Pt electrode.

**FIGURE 7:**
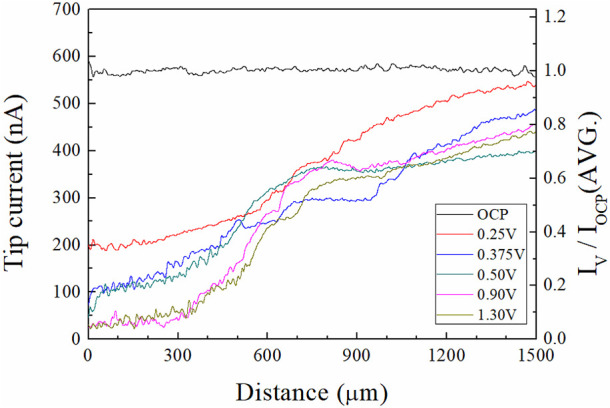
Profiles of water electrolysis currents measured by SECM in 85% phosphoric acid **(left)** tip currents, and **(right)** I_V_/I_OCP_(AVG.) along the tip distance from the Cu substrate. I_V_ and I_OCP_ mean the tip currents at the applied potential “V” and OCP of the Cu substrate. The measurement system was illustrated in Figure 2.

First, the potential to the Cu substrate was applied for 60 s (the preconditioning described earlier). Then the potential to the Pt tip was applied and the current of the Pt tip were measured with movements from the surface of the Cu substrate to the bulk electrolyte. The two water electrolysis profiles from this experiment are presented in Figure 7. The tip current-tip distance and the I_V_/I_OCP_(Avg.)-tip distance profiles are shown in the left side and right side of Figure 7, respectively. The I_V_/I_OCP_(Avg.)-tip distance is the ratio of the tip current at applied potential “V” on the Cu substrate and the average tip current at the open circuit potential (OCP) of the Cu substrate.

These profiles revealed that the tip current profile varied with the potential of the Cu substrate. At the OCP of the Cu substrate, the current was almost constant, regardless of the position of the tip electrode, because the water acceptors were not consumed for the Cu dissolution reaction. As the ECP potentials on the Cu substrate were increased, the tip currents were decreased; tip currents at the Cu surface (distance between the Pt tip and the Cu substrate: 1 μm) were 590 nA, 200 nA, 72 nA, 45 nA, 23 nA, and 21 nA when the ECP potential was OCP, 0.25, 0.375, 0.50, 0.90, and 1.30 V, respectively. The tip currents increased when the Pt tip moved toward the bulk electrolyte direction at all anodic potentials. These results indicated that the quantity of the water acceptors was affected by the ECP potentials because the magnitude of the tip current is proportional to the quantities of water acceptors. Therefore, profiles were evidence of the water acceptor diffusion mechanism.

**FIGURE 8:**
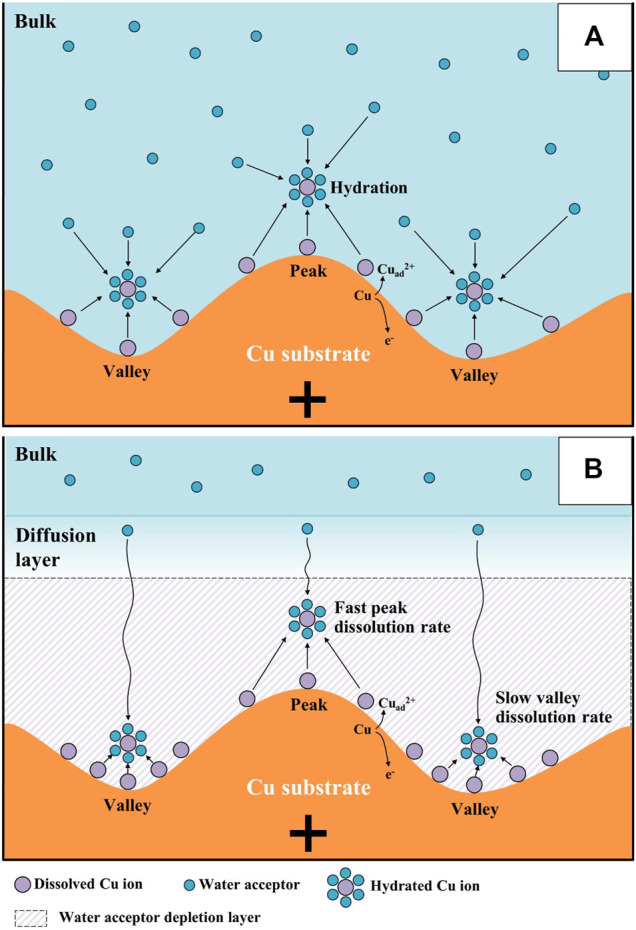
Mechanism scheme of Cu electrochemical polishing: **(A)** initial state, and **(B)** steady state.

Using these water electrolysis profiles and the evidenced mechanism of water acceptor diffusion, the results of LSV and EIS can be also explained. In the LSV in Figure 3, the polishing phenomena occurred at the peak potential of 0.375 V. This LSV behavior might seem to be anodic passivation behavior, but this peak occurred due to the depletion of the water acceptors. Additionally, the polishing phenomena were observed after the potential region at which depletion and diffusion of the water acceptors occurred on the Cu surface; therefore, the diffusion of the water acceptor was closely related with the Cu polishing effects. The results of the Nyquist plots (Figure 6) can be also explained by water acceptor diffusion. In the high-frequency region, the C_dl_ was decreased and the R_p_ was increased as the ECP potential was increased, because the thickness of the diffusion layer of the water acceptor was increased as the ECP potential was increased. Therefore, the difficulty of the Cu dissolution reaction was increased due to the insufficient amount of water acceptors. The presence of the vertical line in the low-frequency region indicates that these conditions correspond with the FSW diffusion condition wherein the viscous diffusion layer and the water acceptors were matched with the limited diffusion layer and the water-limited electroactive substance, respectively. Therefore, the FSW diffusion behavior was caused by this significant and viscous diffusion layer of the water acceptors. These novel electrochemical measurements revealed the Cu ECP mechanism scheme, which is presented in Figure 8. In the initial state of Cu ECP (Figure 8A), when a potential above 0.375 V was applied to the Cu substrate, significant Cu dissolution reactions occurred in both protrusions and the valleys of Cu substrate because many water acceptors hydrated the Cu ions. However, the concentration of the water acceptors rapidly decreased on the Cu surface as the potential was applied; the water acceptors were eventually depleted by hydration for the Cu ions, and the Cu dissolution reaction was governed by a mass transfer control reaction of the water acceptors. Therefore, the water acceptors were preferentially diffused to the surface protrusions, which were more rapidly dissolved than the valleys (Figure 8B). As a result of the rapid dissolution rate at the protrusions, the Cu surface was flattened.

## Conclusion

We successfully identified the mechanism of Cu ECP using various standard electrochemical analysis methods as well as our novel electrochemical analysis method. From the LSV analysis, the Cu polishing effect was observed when the potential was higher than 0.375 V. Anodic potentials were selected for EIS and SECM analyses. The low-frequency region of the resulting Nyquist plots indicated that the diffusion of the water acceptors was related to the Cu polishing phenomena in EIS analyses. Then the profiles of water electrolysis currents and distance enabled the observation of the diffusion of water acceptors for the first time from SECM analyses. These profiles were evidence that the water acceptor mechanism is most likely to explain the polishing phenomena. Knowing the exact mechanism of Cu ECP will be helpful for new interpretations, designs, and applications of ECP.

## Data Availability

The original contributions presented in the study are included in the article/supplementary files, further inquiries can be directed to the corresponding authors.

## Abbreviations

ECP, electrochemical polishing; EIS, electrochemical impedance spectroscopy; LSV, linear scan voltammetry; SEM, scanning electron microscopy; SECM; scanning electrochemical microscopy; FSW, finite space Warburg; OCP, open circuit potential.
